# Self-Organizing Maps to Multidimensionally Characterize Physical Profiles in Older Adults

**DOI:** 10.3390/ijerph191912412

**Published:** 2022-09-29

**Authors:** Lorena Parra-Rodríguez, Edward Reyes-Ramírez, José Luis Jiménez-Andrade, Humberto Carrillo-Calvet, Carmen García-Peña

**Affiliations:** 1 Research Department, Instituto Nacional de Geriatría, Mexico City 10200, Mexico; 2 Facultad de Ciencias, Universidad Nacional Autónoma de México, Mexico City 04510, Mexico; 3 Centro de Ciencias de la Complejidad, Universidad Nacional Autónoma de México, Mexico City 04510, Mexico; 4 Centro de Investigación e Innovación en Tecnologías de la Información y Comunicación, INFOTEC, Mexico City 14050, Mexico

**Keywords:** self-organizing maps (SOM), artificial neural network analysis, body composition and physical performance tests, older adults

## Abstract

The aim of this study is to automatically analyze, characterize and classify physical performance and body composition data of a cohort of Mexican community-dwelling older adults. Self-organizing maps (SOM) were used to identify similar profiles in 562 older adults living in Mexico City that participated in this study. Data regarding demographics, geriatric syndromes, comorbidities, physical performance, and body composition were obtained. The sample was divided by sex, and the multidimensional analysis included age, gait speed over height, grip strength over body mass index, one-legged stance, lean appendicular mass percentage, and fat percentage. Using the SOM neural network, seven profile types for older men and women were identified. This analysis provided maps depicting a set of clusters qualitatively characterizing groups of older adults that share similar profiles of body composition and physical performance. The SOM neural network proved to be a useful tool for analyzing multidimensional health care data and facilitating its interpretability. It provided a visual representation of the non-linear relationship between physical performance and body composition variables, as well as the identification of seven characteristic profiles in this cohort.

## 1. Introduction

It has been shown that among older adults, physical performance, including walking speed, grip strength, and balance, are significant predictors of adverse health events such as disability [1,2,3,4,5], hospitalization [6], and mortality [7,8,9,10]. These three variables of physical performance are also related to body composition, particularly lean mass and fat percentage [11,12,13,14].

A wide variety of tests and tools are now available for the characterization of physical performance and body composition; however, they are based on cutoff points that depend on the measurement technique and the availability of reference studies and populations [15]. Although the recommended approaches for measurements of walking speed, handgrip strength, balance, appendicular lean mass, and the fat percentage between populations are similar, the cutoff values of these measurements in different populations may differ because of sex-based differences, ethnicities, body size, lifestyles, and cultural backgrounds. Furthermore, in some regions, because of the distinct states of aging, not all countries use the same age cutoff to define elderly populations [16].

Therefore, it is necessary to explore alternatives not based on pre-established cutoff points since the effective classification of physical health status in older adults is possible only if a set of potential explanatory variables are considered. To analyze multivariate data is a complex problem for which several mathematical techniques have been developed, including principal component analysis, K-means, and other statistical or machine learning algorithms. Self-organizing neural networks based on an unsupervised learning paradigm [17] combined with a hierarchical clustering algorithm have proved to be useful for automatically analyzing and classifying entities characterized by multivariate data [18]. This technology is effective in classifying multidimensional data and automatically provides Self-Organizing Maps (SOM), which visually represent data and the knowledge obtained by the mathematical computations in a low-dimensional space. Therefore, these maps enhance interpretability and communicability.

Since humans cannot visualize high-dimensional data, SOM neural networks have been used for many applications, such as the generation of feature maps, pattern recognition, and classification [19]. This technique has received attention in epidemiology with applications involving the clustering of patients with insulin resistance syndrome [20], breast cancer patients [21], dengue patients [22], patients with the temporomandibular joint disorder [23], risk groups in child patients under six months of age [24], lifestyle patterns [25], macular morphologic patterns [26], etc.

The purpose of this paper is to show how the SOM neural network can help to develop, classify, and compare profiles of older adults based on age, physical performance tests (walking speed, handgrip strength, and balance), and body composition (appendicular lean mass and fat percentage). This multidimensional approach provides maps depicting a set of clusters that qualitatively characterize groups of older adults sharing similar profiles of body composition and physical performance.

## 2. Materials and Methods

### 2.1. Study Population and Design

This study is a cross-sectional analysis of data from the 3 Ollin (a compound from Nahuatl: Yei-Three and Ollin-Movement) Project of the National Institute of Geriatrics in Mexico City (Instituto Nacional de Geriatría), a cohort of community-dwelling adults from Mexico City. The objective of the 3 Ollin Project is to develop technologies and techniques for the analysis of physical performance tests and the assessment of risk factors in the elderly. Individuals were recruited by convenience sampling from groups of pensioners from the National Autonomous University of Mexico (UNAM), physical therapy clinics, church groups, and other community programs who were invited to take part in the cohort through informative talks and brochures. People eligible to participate in the study were those (1) who were able to mobilize with or without assisting devices and (2) independent or with low dependency who scored 60 points or more in the Barthel Index for Activities of Daily Living (ADLs). Those who were institutionalized, with contraindications to perform aerobic or physical resistance activities of moderate or intense intensity, with musculoskeletal diseases, severe cognitive impairment or diagnosis of dementia, who had any acute or chronic condition, or any individual that in the judgment of the medical staff, could affect the ability to complete the physical performance tests, were excluded. Written informed consent was obtained from all participants before beginning any test.

The study evaluated participants in two time periods. The first period consisted of the assessment of individuals from July 2017 to January 2018. In the second period, from January 2019 to March 2019, new persons were added to the cohort, and a proportion of individuals who had participated in the first period were reevaluated. All participants attended the Functional Evaluation Research Laboratory of the National Institute of Geriatrics and were evaluated by medical staff, composed of geriatricians, general practitioners, physical therapists, and nutritionists. For the purpose of this analysis, only participants with the first evaluation during the whole duration of the study were included.

### 2.2. Variables

Sex was used as a dichotomic variable (man or woman). Age in years, gait speed in m/s [27], handgrip strength in kg [28], one-legged stance in s [29], lean appendicular mass percentage, fat percentage, height in m, and body mass index in kg/m^2^ were analyzed as continuous variables. Gait speed was recorded from a 6 m usual pace walk in the GAIT Rite (platinum 20, instrumented walkway 204 × 35.5 × 0.25 inches, sample rate 100 Hz). A hand dynamometer (JAMAR Hydraulic Hand Dynamometer, Model J00105, Lafayette Instrument, Lafayette, IN, USA) was used to measure grip strength. Three measurements were taken from each side, and the highest of all was considered. In order to assess static balance, the 4-Stage Balance Test was performed on each subject using a balance platform (Balance System SD Operational/Service Manual; Biodex Medical Systems) by asking each individual to perform parallel, semi-tandem, tandem, and one-legged stance. If the participant could hold the position for ten seconds without moving their feet or needing support, the evaluators proceeded to the next position; if not, the test was stopped. If the participants reached the unipodal stage, they were asked to maintain the position for as long as they could, up to a maximum time of 45 s.

Body composition was measured by dual-energy X-ray absorptiometry (DXA) (Hologic Discovery-WI; Hologic Inc., Bedford, MA, USA) [30]. Total fat (in kg and %), total lean mass (kg), appendicular (arms and legs) lean mass (kg), and body mass index (kg/m^2^) were obtained through the total body scan. Anthropometry was determined following validated methodology and by previously standardized personnel.

Other variables considered were the presence/absence of geriatric syndromes: cognitive impairment (MMSE score 20–23 if education ≥ 5 years, 17–19 if education 1–4 years, ≤16 if education < 1 year [31,32]); activities of daily living dependency (Barthel Index ≤ 90 [33]); instrumental activities of daily living dependency (Lawton Instrumental Activities of Daily Living Scale score ≤ 4 for men and ≤7 for women [34]); depression (7-item Center for Epidemiologic Studies Depression Scale Short Form (CES D-7) score ≥ 5 [35]); fear of falling (FES-I score ≥ 23 [36]); and falls in the previous year of the study obtained by self-report. 

Self-report was used to understand the number of years of education completed and was used as a continuous variable and as a categorical variable with five groups (no education, elementary school, high school, bachelor’s degree, and postgraduate studies).

Finally, presence/absence of comorbidities and number of specific comorbidities were inquired by self-report. The comorbidities were myocardial infarction (MI), congestive heart failure (CHF), cerebrovascular accident (CVA), chronic obstructive pulmonary disease (COPD), arthritis, peptic ulcer disease (PUD), liver disease, diabetes, hemiplegia, chronic kidney disease (CKD), cancer, AIDS, peripheral vascular disease (PVD), and hypertension (HTN).

Diagnosis of osteopenia or osteoporosis was obtained through the DXA (T-score for bone mineral density at the femoral neck, proximal femur, lumbar spine, or whole-body T-score (DXA) ≤ −1.0 SD [37]).

For this analysis, only individuals aged 60 years and older who completed all the physical performance tests were included.

### 2.3. Statistical Analysis

A descriptive analysis was performed for individuals divided by sex. Continuous variables are presented as means and standard deviations, while categorical variables are expressed as number and percentage. A Kolmogorov–Smirnov test was used to assess the normality of the continuous variables, a Leven’s test was used to test the homogeneity of variances, and the differences between means for men and women were tested using a *t*-test with equal variances or unequal variances for normal variables or a Mann-Whitney test for non-parametric variables. Comparisons of men and women were estimated through a χ^2^ test for categorical variables.

A method that combines a SOM neural network and a hierarchical clustering algorithm was used to address the problem of multidimensionally comparing and grouping older adults. In a nutshell, the SOM neural network is modeled as a two-dimensional hexagonal grid [17]. Each hexagon represents an artificial neuron and, at the same time, a location where data points can be mapped. The final (self-organized) map is the result of the neural network iterative training process, by which the network adapts and projects similar multidimensional data into close locations (hexagons) in the map. This no-linear projection provides a visual representation of the multidimensional data distribution in 2D cartography [38]. A brief description of this technology is provided in Appendix A. The software tool LabSOM that was used in this study implements this method and can be obtained freely from the web page referred in [39].

Compared with other multidimensional data analysis techniques (K-means, multidimensional scaling, principal component analysis, etc.), this method excels due to its interpretability advantage and friendly visualization resources that serve to fully inform the characteristics and differences among clusters and data.

Two visualization sceneries were used: (1) A clusters map that visually depicts the identified groups and (2) A set of heat maps (one map for each variable) that allow us to characterize the physical performance profiles of the participants.

Each identified cluster is labeled with a number and colored as the test results worsen. Variable heat maps are colored according to a chromatic scale, ranging with the highest values in green, lowest in red, and yellow for intermediate values. To determine the clusters’ characteristics, we look up the colors in the same zone but on the heat maps. The spatial distribution of clusters also obeys profile similarity. Thus, two adjacent clusters are more similar than those that are not adjacent.

The neural network multidimensional analysis was divided by sex, and six variables were used to determine the physical profile of each participant: age, gait speed over height (Gait/height), grip strength over body mass index (Grip/BMI), one-legged stance (Balance), lean appendicular mass percentage (LAM%), and fat percentage (Fat%). Gait speed was divided over height, and grip strength was divided over BMI since taller stature is associated with faster gait speed [40] and handgrip strength is correlated with BMI [41]. All variables were standardized (rescaled to have a mean of zero and a standard deviation of one) so that they are dimensionless and have the same scale. Thus, physical profiles are modeled as vectors in a six-dimensional space that the neural network has to compare and classify.

A Shapiro–Wilk test was used to assess the normality of the distribution of the variables in each cluster (Age, Gait/height, Grip/BMI, Balance, LAM%, and Fat%). The differences between means and distributions of the variables were tested using: (1) An ANOVA with post hoc Tukey’s test for the variables that resulted in being normal with equal variances; (2) A Welch ANOVA with post hoc Games–Howell test for the variables that resulted in being normal with unequal variances; (3) A Kruskal–Wallis with Dunn post hoc test for the variables that were not normally distributed. A matrix was constructed in order to visualize in which clusters the variable means have a statistically significant difference. Complete results can be seen in Appendix B.

A multinomial logistic regression model (univariate analysis) was applied to determine the relationship between the cluster classification and the conditions that were not included in the NNA (presence of comorbidities, cognitive impairment, dependence, depression, fear of falling, and years of education completed) and a Poisson Regression was used to assess whether the cluster classification influences the number of comorbidities obtained on each cluster. Appendix C contains the complete results of all variables where this analysis was statistically significant. The descriptive and inferential analyses were performed with the statistical package software IBM SPSS Statistics (version 17.0, IBM, Chicago, IL, USA).

## 3. Results

A total of 620 individuals were included in the 3 Ollin cohort, 564 aged 60 years and older and 56 aged younger than 60 years. For the purpose of this paper, only the 564 individuals aged 60 years and older were considered in the analysis, and two individuals were discarded because they did not complete the physical performance and body composition tests. The mean age of the studied population was 71.2 ± 7.0 years, with women comprising 73.3% of the total cohort.

The characteristics of the study population, such as demographics, comorbidities, mental status, body composition, dependency, mobility, balance, and strength, are shown in Table 1. Statistically significant differences (*p* < 0.001) between women and men in strength, body composition, dependency on instrumental activities of daily living, and fear of falling were found. Prevalence of myocardial infarction and diabetes was higher in men, while the prevalence of arthritis, peripheral vascular disease, and osteopenia or osteoporosis were higher in women (*p* < 0.01).

**Figure 1:**
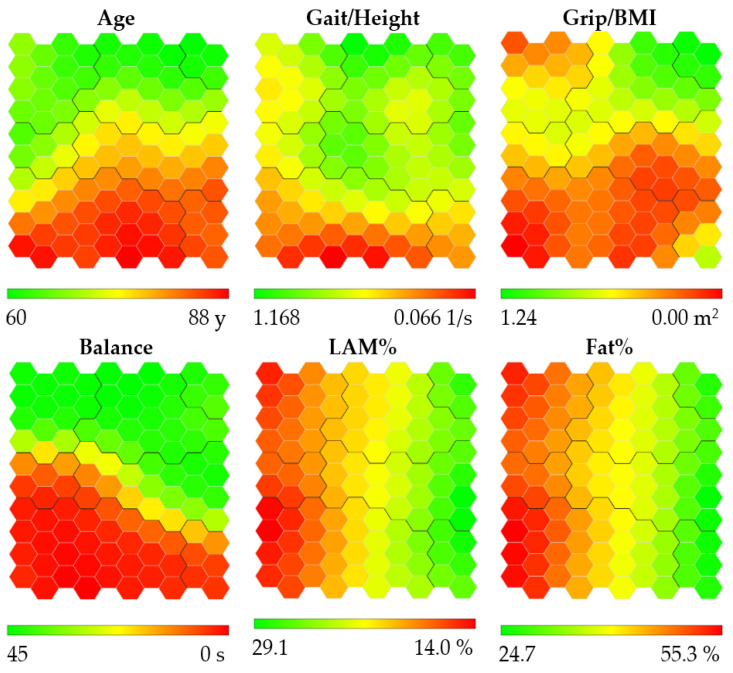
Heat maps obtained for women. Abbreviations refer to: gait speed/height (Gait/Height), grip strength/body mass index (Grip/BMI), one-legged stance (Balance), lean appendicular mass percentage (LAM%), and fat percentage (Fat%). Heat maps are colored according to a chromatic scale. The best physical performance and body composition values are colored in green (lower Age and Fat%, and higher Gait/Height, Grip/BMI, LAM% and Balance), worst values are colored in red (higher age and Fat%, and lower Gait/Height, Grip/BMI, LAM% and Balance), and intermediate values are colored in yellow and orange. The contours of the obtained clusters are visible on each map.

The heat maps obtained with the SOM for women are presented in Figure 1. These maps are colored according to a chromatic scale: the best physical performance and body composition values are colored in green (lower Age and Fat%, and higher Gait/Height, Grip/BMI, Balance, and LAM%), worst values are colored in red (higher Age and Fat%, and lower Gait/Height, Grip/BMI, Balance, and LAM%), and intermediate values are colored in yellow and orange. Thus, as shown in Figure 1, colored in green are the youngest women with the best balance located at the top of the maps, the fastest and strongest women are located at the top right of the maps, and the women with a higher percentage of lean appendicular mass and lower fat percentage are located at the right of the maps. 

**Figure 2:**
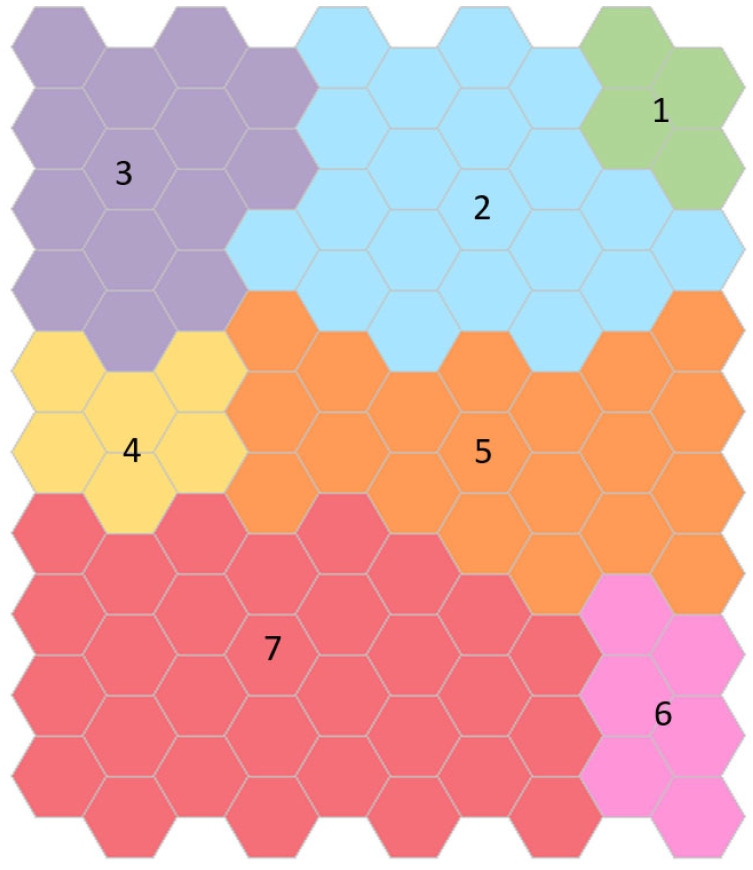
Clusters map obtained for women. Each identified cluster is labeled with a number from one to seven and colored as the test results worsen: green, blue, purple, yellow, orange, pink, and red, respectively. Cluster 1, colored in green, contains the youngest and healthiest women. Women included in Cluster 7, colored in red, are the oldest, with the lowest gait speed and strength, the worst balance, the lowest muscle mass, and the highest fat mass.

The cluster map obtained for women is shown in Figure 2. It is important to note that the farther the neurons are on the map, the more different the people are within them. Hence, women in Cluster 1 are more similar to women in the adjacent Cluster 2 and are more different than women in Cluster 7. Each identified cluster was labeled with a number as the test results worsened. Thus, the clusters were placed in ascending order as age increases, physical performance decreases, and body composition worsens, with some exceptions: women in Cluster 4 are stronger than women in Cluster 3; women in Cluster 5 have better body composition than women in Clusters 2 to 4, are faster than women in Cluster 3, and have better balance than women in Cluster 4; women in Cluster 6 have better body composition than women in Clusters 2 to 5; women in Cluster 7 have better body composition than women in Cluster 3 and have more muscle than women in Cluster 4. 

Cluster 1 contains the youngest (62.9 ± 2.2 years) and healthiest women, those with higher gait speed, handgrip strength, good balance, and the best body composition, higher lean appendicular mass, and lower fat mass. Only 20 women were located in this cluster. On the other hand, women included in Cluster 7 are the oldest (77.1 ± 5.5 years), with the lowest gait speed and strength, the worst balance, lowest muscle mass, and highest fat mass. The descriptive of the variables of the neural network analysis and those that were associated with the cluster classification for women are shown in Table 2.

Statistically significant differences (*p* < 0.05) between pairs of clusters for the SOM variables and positive or negative mean differences between variables can be seen in Table 3 (full results when comparing the difference in means across the variables within the clusters can be found in Table A1). Statistically significant differences were found in almost all clusters and within all variables when comparing women in Cluster 7.

Univariate multinomial logistic regression between the cluster classification and CI, ADLD, FF, EDUC, PVD, and HTN showed statistically significant results. Poisson regression was applied to the number of comorbidities (NUMCOM). Table 4 presents significant relative risk ratios for the presence of adverse clinical conditions when comparing pairs of clusters (full results RRR, *p* values, and 95% confidence intervals for all cluster comparisons are specified in Table A3, Table A4, Table A5 and Table A6).

Being grouped in Cluster 7 was associated with an increased likelihood of exhibiting peripheral vascular disease, hypertension, fear of falling, cognitive impairment, dependence, and presenting more comorbidities than women in other clusters. For example, they were 10.2, 4.8, and 2.8 times more likely to present hypertension than women in Clusters 1, 2, and 3, respectively. On the other hand, increasing education was associated with a decreased likelihood of being in Cluster 7 when compared with other clusters (Table 4).

**Figure 3:**
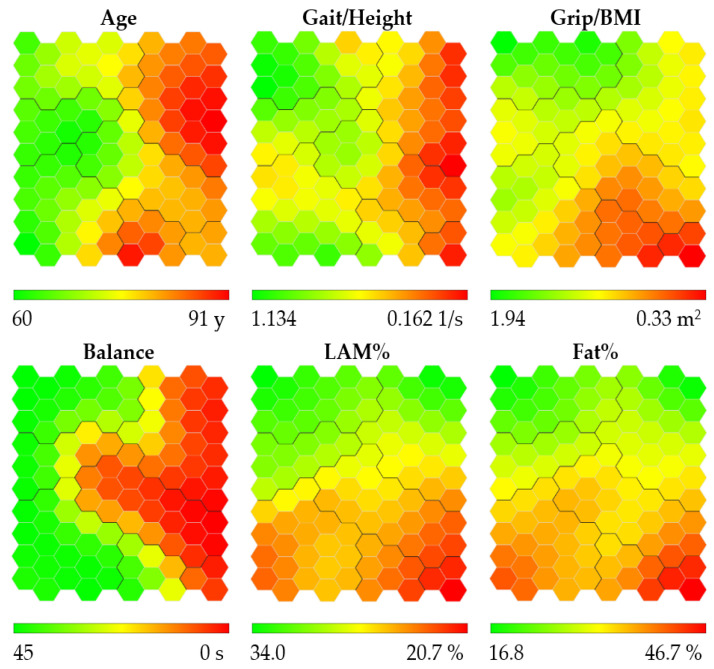
Heat maps obtained for men. Abbreviations refer to: gait speed/height (Gait/Height), grip strength/body mass index (Grip/BMI), one-legged stance (Balance), lean appendicular mass percentage (LAM%), and fat percentage (Fat%). Heat maps are colored according to a chromatic scale. The best physical performance and body composition values are colored in green (lower Age and Fat%, and higher Gait/Height, Grip/BMI, LAM% and Balance), worst values are colored in red (higher age and Fat%, and lower Gait/Height, Grip/BMI, LAM% and Balance), and intermediate values are colored in yellow and orange. The contours of the obtained clusters are visible on each map.

Similarly to the case of women, the heat maps were obtained for men, as shown in Figure 3. The youngest men are located in the middle left of the map, the fastest and strongest men are located at the top left of the map, the men with the best balance are located at the left of the map, and the men with the highest percentage of lean appendicular mass and least fat percentage are located at the top of the map. 

**Figure 4:**
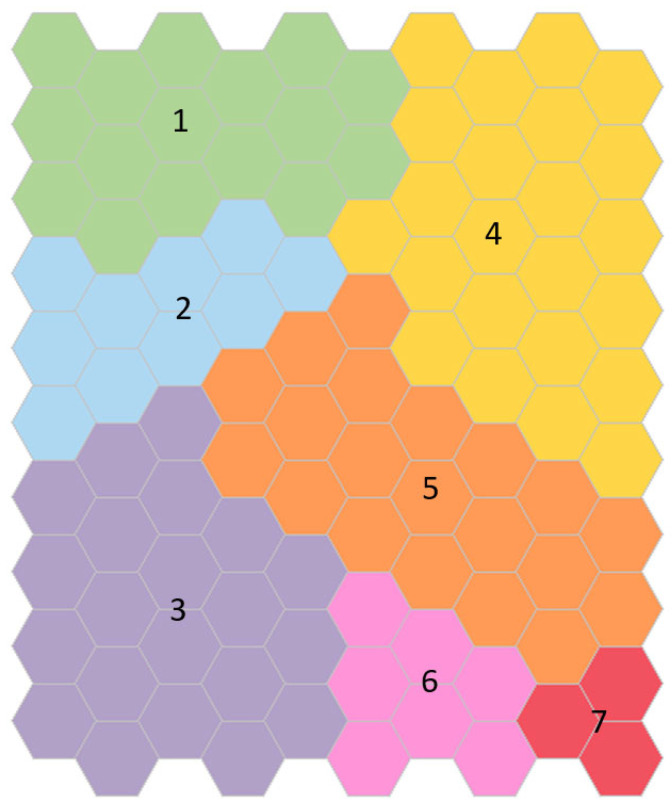
Clusters map obtained for men. Each identified cluster is labeled with a number from one to seven and colored as the test results worsen: green, blue, purple, yellow, orange, pink, and red, respectively. Cluster 1, colored in green, contains the youngest and healthiest men. Men included in Cluster 7, colored in red, are the oldest, with the lowest gait speed and strength, the worst balance, the lowest muscle mass, and the highest fat mass.

Figure 4 shows the cluster map obtained for men. The clusters were labeled from one to seven according to the worsening of the test results. Some exceptions should be noted: men in Cluster 2 are younger than men in Cluster 1; men in Cluster 3 have worse body composition than men in Cluster 4; men in Cluster 4 are older and slower than men in Cluster 5; men in Cluster 6 have better balance than men in Clusters 4 and 5.

Men in Cluster 1 are in the middle of the age scale (70.0 ± 5.1 years); however, they have better physical performance and body composition than men in other Clusters, and, as mentioned before, they even have better results than younger men in Cluster 2. A complete description of the clusters is shown in Table 5.

Differences between pairs of clusters for the SOM variables and positive or negative mean differences between variables can be seen in Table 6 (full results for testing differences in means across the variables within the clusters can be found in Table A2). The five men included in Cluster 7 have the lowest gait speed, strength, and balance and have the lowest muscle mass and highest fat mass. Statistically significant differences were found between this cluster and all the other clusters and within all variables.

On the other hand, after applying the logistic regression analysis, statistically significant results were found for PVD, HTN, and NUMCOM, as shown in Table 7 (full results RRR, *p* values, and 95% confidence intervals for all cluster comparisons are specified in Table A7 and Table A8). Being grouped in Clusters 4, 5, 6, and 7 was associated with an increased likelihood of exhibiting peripheral vascular disease and hypertension and presenting more comorbidities than men in the first Clusters 1, 2, and 3. For example, men in Cluster 4 were 5.0, 6.7, and 4.7 times more likely to present hypertension than men in Clusters 1, 2, and 3, respectively. 

## 4. Discussion

To the best of our knowledge, this is the first study that analyses the competing effect between physical performance and body composition for older women and men using a SOM approach. The physical profiles were divided by sex, and the multidimensional analysis included age, gait speed over height, grip strength over body mass index, one-legged stance, lean appendicular mass percentage, and fat percentage. Using the SOM neural network, seven profile groups for older men and women were obtained. With this method, older adults were categorized according to their multidimensional profile instead of applying univariate criteria.

The heterogeneity of health and function among older adults cannot be explained by comorbidity alone [42]. As a result, efforts have focused on capturing other factors determining health in later life, and new late-life syndromes such as frailty and sarcopenia have been defined. However, there is an absence of an internationally accepted definition of these syndromes, and their prevalence is known to vary noticeably, depending on the studied population, measurements, and cutoff points used [15,43,44]. The advantages of the multidimensional SOM approach are that it uses several objective variables; it does not define cutoff points; it provides useful visual outputs; it is suitable to study large groups since the neural network training is not computationally expensive, and the more people are included in the training, the more accurate the clustering will be. Additionally, the heat maps associated with the clusters map provide a friendly way to visually characterize and compare the seven obtained profiles.

Customarily regression approaches, such as linear, logistic, or proportional hazards models, have limitations when analyzing correlated variables due to the collinearity between them. The relationship between physical performance and body composition has been traditionally approached through these analyses [12,45,46,47]. Results from most studies indicate that an increase in fat or a decrease in muscle mass causes greater functional disability and lower physical performance. However, these approaches have also led to inconsistent findings, usually attributed to not considering interactions with other variables or the establishment of reference values for each variable that have to be validated across age, sex, and race [47].

Traditional multivariate analysis, such as factor analysis and principal component analysis, aims to group by similarities across variables to remove collinearity, decrease variable redundancy, and help reveal the underlying structure of the input variables in a data set. There are few studies centered on older adults that examined the combined effects of several objective variables at the same time. For example, strength, physical function, muscle and adiposity characteristics, and risk of disability in older adults were studied in [48]. Factor analysis reduced these variables into a smaller number of components. In [49] the association between ethnicity, sociodemographic, health, and lifestyle factors, and physical performance were analyzed by a multivariable linear regression to identify factors associated with upper body strength and mobility. An artificial neural network was used to study which general characteristics and health data were the best to predict frailty [50]. In [51], the relationship between body composition and cognitive functioning in an elderly people’s sample was analyzed. Correlation analysis, linear regression, and cluster analysis were carried out to analyze the relationships between the different measures. Thus, the multivariate analysis of these studies searched for associations, groupings, or relationships between variables and risk factors, not between individuals. In [51], k-means cluster analysis was performed to obtain only two groups based on body composition, but their visualization resources were limited.

Visual analysis is a complementary tool to obtain objective conclusions from the analyzed data [52,53]. In our study, the visual analysis aided the profile characterization and comparison process and the identification of relations between variables. The SOM neural network, together with PCA and MDS methods, are among the most employed tools to visualize multidimensional data [53]. However, the several visualizations and sceneries produced by the SOM make it better suited for visual analysis and cluster analysis. Furthermore, when having large amounts of data, the SOM neural network is a better option because it has better scalability properties.

Cluster analysis aims to group individuals by similarities across observations, such that persons in the same group are as similar to each other as possible and individuals in different groups are as different from each other as possible [54]. Thus, in our study, it was possible to identify older adults with similar features, while at the same time, differences between groups were found. Additionally, the color coding of the heat maps helps to visualize the range and relationship between variables. These results could help to design resistance, balance, and nutritional interventions to prevent, delay, or reverse adverse outcomes and to improve functional ability in older adults according to their specific characteristics and needs [55,56]. Furthermore, this method allows new individuals to be classified within the cluster structure, but more research is needed to derive models that predict adverse outcomes.

Our study also considered the association of the cluster assignation with several risk factors: cognitive impairment, dependence, depression, fear of falling, diagnosis of osteopenia or osteoporosis, falls in the previous year of the study, number of years of education completed, myocardial infarction, congestive heart failure, cerebrovascular accident, chronic obstructive pulmonary disease, arthritis, peptic ulcer disease, liver disease, diabetes, hemiplegia, chronic kidney disease, cancer, AIDS, peripheral vascular disease, and hypertension. 

The variables with statistically significant relationships with the cluster classification for both men and women were the presence of hypertension, peripheral vascular disease, and the number of comorbidities present. For women, cognitive impairment, fear of falling, dependence on activities of daily living, and years of education were also significant variables associated with the clustering.

The relationship between advanced age, muscle strength, physical performance, low muscle mass, and obesity with hypertension [57,58,59,60], peripheral arterial disease [61,62], and comorbidity [63,64] has been studied in depth, and the importance of diagnosing and intervention of poor physical performance and body composition due to its association with diverse adverse health outcomes such as cognitive impairment, loss of dependence, falls, fractures, and mortality [64,65] has been emphasized. 

When comparing women in Cluster 7 with women of the same age in Cluster 5 and younger women in Clusters 2 and 3, women in Cluster 7 are more likely to present cognitive impairment, fear of falling, dependence, and fewer years of education than women in these other clusters. Years of education is a critical component of health, and a shorter duration of education has been associated with low muscle mass and strength [66,67]. 

This study has some limitations. First, as we excluded older adults with high dependency, severe cognitive impairment, and with musculoskeletal diseases, the cluster classification does not include individuals with these characteristics. Second, the cross-sectional analysis prevents establishing a causal relationship between variables; therefore, a longitudinal follow-up of the participants is needed to determine a temporal association and to further validate the cluster classification. Third, our study did not assess other factors associated with physical performance and body composition, including genetic determinants, physical activity, diet, and environmental and social factors. Fourth, a larger sample of men is needed in order to have more variability and representability for this group. 

## 5. Conclusions

There is a growing consensus that interventions targeting mobility, strength, balance, nutrition, and physical activity may offer the best opportunity to prevent, delay, or reverse adverse outcomes and to improve functional ability in older adults [55,56]. However, in order to enhance the impact of these interventions, they should be tailored and supervised according to the specific characteristics and needs of older adults. The neural network approach presented in this study provides a multidimensional perspective to screen and group individuals with similar body composition and physical performance profiles.

The SOM neural network has proved to be a convenient tool for analyzing multidimensional health care data. This method facilitated the interpretability of high-dimensional data through heat and cluster maps providing a visual representation of the non-linear relationships between physical performance and body composition variables and deriving seven characteristic profiles for the female and male populations analyzed in this study. These results open a new horizon into the research, characterization, and design of interventions for the identified profiles.

## Figures and Tables

**Table 1:** Characteristics of older adults.

	Total *n* = 562 Mean ± SD or *n* (%)	Women *n* = 412 Mean ± SD or *n* (%)	Men *n* = 150 Mean ± SD or *n* (%)	*p*-Value
**Neural network analysis physical profile**				
Age, y	71.2 ± 7.0	71.0 ± 7.1	71.7 ± 6.9	0.296
Gait speed over height (Gait/Height), 1/s	0.654 ± 0.163	0.657 ± 0.165	0.645 ± 0.158	0.456
Grip strength over body mass index (Grip/BMI), m^2^	0.711 ± 0.322	0.583 ± 0.215	1.062 ± 0.307	<0.001
One-legged stance (Balance), s	25.7 ± 19.2	25.4 ± 19.3	26.6 ± 19.2	0.321
Lean appendicular mass percentage (LAM%), %	23.1 ± 3.4	21.6 ± 2.2	27.1 ± 2.6	<0.001
Fat percentage (Fat%), %	39.5 ± 6.8	42.3 ± 5.0	31.9 ± 5.0	<0.001
Height, (m)	1.56 ± 0.09	1.53 ± 0.07	1.67 ± 0.07	<0.001
Weight, (kg)	67.4 ± 13.0	64.3 ± 11.4	75.9 ± 13.3	<0.001
Body mass index (BMI), kg/m^2^	27.5 ± 4.3	27.6 ± 4.5	27.2 ± 4.0	0.499
Gait speed, cm/s	102.4 ± 26.4	100.4 ± 26.1	107.7 ± 26.7	0.004
Grip strength, kg	19.1 ± 8.2	15.7 ± 5.3	28.4 ± 7.7	<0.001
Cognitive impairment (CI)	76 (13.5)	56 (13.6)	20 (13.4)	0.959
Activities of daily living dependence (ADLD)	63 (11.2)	48 (11.7)	15 (10.0)	0.577
Instrumental activities of daily living dependence (IADLD)	99 (17.6)	94 (22.9)	5 (3.3)	<0.001
Depression	187 (33.3)	149 (36.3)	38 (25.5)	0.017
Fear of falling (FF)	336 (59.9)	268 (65.2)	68 (45.3)	<0.001
Fell last year	241 (42.9)	187 (45.4)	54 (36.0)	0.047
**Education (EDUC), y**	13.9 ± 5.5	13.1 ± 5.4	16.0 ± 5.4	<0.001
No education	9 (1.6)	9 (2.2)	0	<0.001
Elementary school	126 (22.4)	102 (24.7)	24 (16.0)	
High school	130 (23.1)	112 (27.2)	18 (12.0)	
Bachelor’s degree	221 (39.3)	148 (35.9)	73 (48.7)	
Postgraduate	76 (13.5)	41 (10.0)	35 (23.3)	
**Comorbidities**				
Myocardial infarction (MI)	46 (8.2)	25 (6.1)	21 (14.0)	0.002
Congestive heart failure (CHF)	11 (2.0)	9 (2.2)	2 (1.3)	0.519
Cerebrovascular accident (CVA)	17 (3.0)	15 (3.6)	2 (1.3)	0.160
Chronic obstructive pulmonary disease (COPD)	33 (5.9)	23 (5.6)	10 (6.7)	0.629
Arthritis	64 (11.4)	56 (13.6)	8 (5.3)	0.006
Peptic ulcer disease (PUD)	233 (41.5)	182 (44.2)	51 (34.0)	0.030
Liver disease	45 (8.0)	34 (8.3)	11 (7.3)	0.717
Diabetes	94 (16.1)	56 (13.6)	38 (25.3)	0.001
Hemiplegia	43 (7.7)	32 (7.8)	11 (7.3)	0.864
Chronic kidney disease (CKD)	6 (1.1)	4 (1.0)	2 (1.3)	0.712
Cancer	69 (12.3)	51 (12.4)	18 (12.0)	0.904
AIDS	0	0	0	-
Peripheral vascular disease (PVD)	252 (44.8)	212 (51.5)	40 (26.7)	<0.001
Hypertension (HTN)	262 (46.6)	194 (47.1)	68 (45.3)	0.712
**Number of comorbidities (NUMCOM)**				0.018
0	67 (12.0)	40 (9.8)	27 (18.1)	
1	156 (27.9)	114 (27.8)	42 (28.2)	
2	140 (25.0)	113 (27.6)	27 (18.1)	
3	100 (17.9)	66 (16.1)	34 (22.8)	
4	64 (11.4)	49 (12.0)	15 (10.1)	
5	20 (3.6)	17 (4.1)	3 (2.0)	
6	9 (1.6)	8 (2.0)	1 (0.7)	
7	3 (0.5)	3 (0.7)	0	
Osteopenia/Osteoporosis	429 (76.3)	337 (81.8)	92 (61.3)	<0.001

There were 1 missing data for women for depression, ADLD, IADLD, FF, CVA, and liver disease, and 2 missing data for number of comorbidities. There were 1 missing data for men for MMSE, depression, CVA, and number of comorbidities. A *t*-test with equal variances was used to compare means between men and women for Gait/height, Fat%, and gait speed. A *t*-test with unequal variances was used to compare means between men and women for Grip/BMI and LAM%. A Mann-Whitney test was used to compare means between men and women for age, balance, height, BMI, grip strength, and years of education. A χ^2^ test was used for categorical variables.

**Table 2:** Characteristics of the seven clusters obtained for women.

Women	Cluster 1	Cluster 2	Cluster 3	Cluster 4	Cluster 5	Cluster 6	Cluster 7
	*n*1 = 20	*n*2 = 81	*n*3 = 72	*n*4 = 25	*n*5 = 68	*n*6 = 17	*n*7 = 129
Age (y)	62.9 ± 2.2	66.2 ± 4.9	66.1 ± 4.2	66.4 ± 4.3	73.3 ± 4.9	76.3 ± 4.8	77.1 ± 5.5
Gait/Height (1/s)	0.804 ± 0.162	0.747 ± 0.140	0.666 ± 0.114	0.666 ± 0.111	0.746 ± 0.127	0.559 ± 0.094	0.536 ± 0.153
Grip/BMI (m^2^)	0.951 ± 0.140	0.781 ± 0.139	0.545 ± 0.166	0.646 ± 0.128	0.524 ± 0.179	0.652 ± 0.215	0.433 ± 0.139
Balance (s)	39.0 ± 12.5	43.0 ± 6.8	42.6 ± 6.1	8.2 ± 6.2	31.7 ± 15.1	7.9 ± 7.2	4.8 ± 6.8
LAM% (%)	24.8 ± 1.4	22.3 ± 1.2	19.9 ± 1.2	20.0 ± 1.0	23.2 ± 1.8	24.6 ± 1.3	20.8 ± 2.0
Fat% (%)	34.6 ± 2.9	40.9 ± 2.8	46.3 ± 2.7	45.8 ± 2.1	38.7 ± 4.2	34.4 ± 3.4	44.3 ± 4.3
CI	1 (5.0)	8 (9.9)	8 (11.1)	2 (8.0)	5 (7.4)	2 (11.8)	30 (23.3)
ADLD	1 (5.0)	3 (3.7)	4 (5.6)	4 (16.0)	5 (7.4)	2 (11.8)	29 (22.5)
FF	5 (25.0)	39 (48.1)	48 (66.7)	16 (64.0)	44 (64.7)	11 (64.7)	105 (81.4)
EDUC (y)	13.9 ± 4.6	13.7 ± 5.9	14.2 ± 5.2	12.8 ± 5.3	14.1 ± 4.2	12.2 ± 6.1	11.6 ± 5.6
PVD	9 (45.0)	40 (49.4)	35 (48.6)	11 (44.0)	25 (36.8)	8 (47.1)	84 (65.1)
HTN	3 (15.0)	22 (27.2)	28 (38.9)	11 (44.0)	40 (58.8)	7 (41.2)	83 (64.3)
NUMCOM							
0	3 (15.0)	14 (17.3)	8 (11.1)	3 (12.0)	7 (10.3)	2 (12.5)	3 (2.3)
1	8 (40.0)	20 (24.7)	25 (34.7)	8 (32.0)	21 (30.9)	5 (31.1)	27 (21.1)
2	4 (20.0)	30 (37.0)	22 (30.6)	3 (12.0)	17 (25.0)	4 (25.0)	33 (25.8)
3	3 (15.0)	13 (16.0)	7 (9.7)	6 (24.0)	9 (13.2)	2 (12.5)	26 (20.3)
4	2 (10.0)	2 (2.5)	6 (8.3)	4 (16.0)	12 (17.6)	2 (12.5)	21 (16.4)
≥5	0	2 (2.5)	4 (5.6)	1 (4.0)	2 (2.9)	1 (6.3)	18 (14.1)

Abbreviations refer to: gait speed/height (Gait/Height), grip strength/body mass index (Grip/BMI), one-legged stance (Balance), lean appendicular mass percentage (LAM%), fat percentage (Fat%), cognitive impairment (CI), activities of daily living dependence (ADLD), fear of falling (FF), years of education completed (EDUC), peripheral vascular disease (PVD), hypertension (HTN), and number of comorbidities (NUMCOM). In Cluster 7 there were 1 missing data for FF, ADLD, and NUMCOM. In Cluster 6 there were 1 missing data for NUMCOM.

**Table 3:** Comparisons between pairs of clusters for women for the variables included in the SOM analysis.

Women		Cluster					
		1					
**Cluster**	**2**	<Grip/BMI					
		<LAM%	**Cluster**				
		>Fat%	**2**				
	**3**	<Gait/height	<Gait/height				
		<Grip/BMI	<Grip/BMI				
		<LAM%	<LAM%	**Cluster**			
		>Fat%	>Fat%	**3**			
	**4**	<Gait/height					
		<Grip/BMI	<Grip/BMI	>Grip/BMI			
		<Balance	<Balance	<Balance			
		<LAM%	<LAM%		**Cluster**		
		>Fat%	>Fat%		**4**		
	**5**	>Age	>Age	>Age	>Age		
				>Gait/height			
		<Grip/BMI	<Grip/BMI		<Grip/BMI		
			<Balance	<Balance	>Balance		
		<LAM%	>LAM%	>LAM%	>LAM%	**Cluster**	
				<Fat%	<Fat%	**5**	
	**6**	>Age	>Age	>Age	>Age		
		<Gait/height	<Gait/height	<Gait/height	<Gait/height	<Gait/height	
		<Grip/BMI					
		<Balance	<Balance	<Balance		<Balance	
			>LAM%	>LAM%	>LAM%	>LAM%	**Cluster**
			<Fat%	<Fat%	<Fat%		**6**
	**7**	>Age	>Age	>Age	>Age		
		<Gait/height	<Gait/height	<Gait/height	<Gait/height	<Gait/height	
		<Grip/BMI	<Grip/BMI	<Grip/BMI	<Grip/BMI	<Grip/BMI	<Grip/BMI
		<Balance	<Balance	<Balance		<Balance	
		<LAM%	<LAM%	>LAM%	>LAM%	<LAM%	<LAM%
		>Fat%	>Fat%	<Fat%		>Fat%	>Fat%

Means comparisons were applied to gait speed/height (Gait/Height), grip strength/body mass index (Grip/BMI), one-legged stance (Balance), lean appendicular mass percentage (LAM%), and fat percentage (Fat%). Only statistically significant results are shown (*p* < 0.05). Each result should be read as follows: The VARIABLE mean for women in Cluster ROW is greater/less (>/<) than the VARIABLE mean for women in Cluster COLUMN. For example: The grip/BMI mean for women in Cluster 2 is less than the grip/BMI mean for women in Cluster 1.

**Table 4:** Comparisons between pairs of clusters for women for conditions not included in the SOM analysis.

Women		Cluster					
		1					
**Cluster**	**2**						
			**Cluster**				
			**2**				
	**3**	6.0 FF	2.2 FF				
				**Cluster**			
				**3**			
	**4**		5.0 ADLD				
		5.3 FF			**Cluster**		
		4.5 HTN			**4**		
	**5**	5.5 FF	2.0 FF				
		8.1 HTN	3.8 HTN	2.2 HTN		**Cluster**	
						**5**	
	**6**	5.5 FF					
							**Cluster**
							**6**
	**7**		2.8 CI	2.4 CI		3.8 CI	
			7.6 ADLD	5.0 ADLD		3.7 ADLD	
		13.7 FF	4.9 FF	2.3 FF	2.6 FF	2.5 FF	
			0.93 EDUC	0.91 EDUC		0.91 EDUC	
			1.2 PVD	2.0 PVD		3.2 PVD	
		10.2 HTN	4.8 HTN	2.8 HTN			
		1.7 NUMCOM	1.6 NUMCOM	1.5 NUMCOM		1.4 NUMCOM	

Significant (*p* < 0.05) relative risk ratios when comparing pairs of clusters. Univariate multinomial logistic regression was applied to cognitive impairment (CI), activities of daily living dependence (ADLD), fear of falling (FF), years of education completed (EDUC), peripheral vascular disease (PVD), and hypertension (HTN). Poisson regression was applied to number of comorbidities (NUMCOM). Each result for the dichotomic variables should be read as follows: Women in Cluster ROW are RRR times more/less (if RRR > 1/if RRR < 1) likely to present CONDITION, than women in Cluster COLUMN. For example: Women in Cluster 7 are 10.2 times more likely to present HTN than women in Cluster 1.

**Table 5:** Characteristics of the seven clusters obtained for men.

Men	Cluster 1	Cluster 2	Cluster 3	Cluster 4	Cluster 5	Cluster 6	Cluster 7
	*n*1 = 27	*n*2 = 21	*n*3 = 29	*n*4 = 31	*n*5 = 27	*n*6 = 10	*n*7 = 5
Age, (y)	70.0 ± 5.1	65.2 ± 3.5	67.9 ± 5.2	78.4 ± 4.7	71.8 ± 5.4	78.5 ± 7.2	75.8 ± 6.4
Gait/Height, (1/s)	0.760 ± 0.151	0.690 ± 0.110	0.708 ± 0.112	0.504 ± 0.139	0.635 ± 0.113	0.640 ± 0.142	0.418 ± 0.124
Grip/BMI, (m^2^)	1.440 ± 0.238	1.106 ± 0.168	1.054 ± 0.222	1.077 ± 0.194	0.888 ± 0.213	0.685 ± 0.193	0.469 ± 0.144
Balance, (s)	44.1 ± 4.8	34.4 ± 15.8	43.5 ± 4.9	8.3 ± 9.1	5.4 ± 5.1	39.1 ± 8.6	5.0 ± 9.1
LAM%, (%)	30.0 ± 1.6	28.7 ± 1.0	25.5 ± 1.4	28.1 ± 2.3	25.5 ± 1.5	24.7 ± 1.3	22.7 ± 1.4
Fat%, (%)	26.4 ± 3.5	30.5 ± 2.5	35.3 ± 2.6	29.5 ± 4.3	34.6 ± 3.0	35.5 ± 3.3	40.6 ± 5.3
PVD	6(22.2)	1(4.8)	5(17.2)	12(38.7)	9(33.3)	6(60.0)	1(20.0)
HTN	8 (29.6)	5 (23.8)	9 (31.0)	21 (67.7)	16 (59.3)	5 (50.0)	4 (80.0)
NUMCOM							
0	4 (15.4)	9 (42.9)	9 (31.0)	2 (6.5)	2 (7.4)	1 (10.0)	0
1	13 (50.0)	7 (33.3)	8 (27.6)	4 (12.9)	8 (29.6)	1 (10.0)	1 (20.0)
2	5 (19.2)	4 (19.0)	5 (17.2)	7 (22.6)	6 (22.2)	0	0
3	2 (7.7)	1 (4.8)	6 (20.7)	10 (32.3)	9 (33.3)	4 (40.0)	2 (40.0)
4	1 (3.8)	0	1 (3.4)	6 (19.4)	2 (7.4)	4 (40.0)	1 (20.0)
≥5	1 (3.8)	0	0	2 (6.4)	0	0	1 (20.0)

Abbreviations refer to: gait speed/height (Gait/Height), grip strength/body mass index (Grip/BMI), one-legged stance (Balance), lean appendicular mass percentage (LAM%), fat percentage (Fat%), peripheral vascular disease (PVD), hypertension (HTN), and number of comorbidities (NUMCOM). There were 1 missing data in Cluster 1 for number of comorbidities.

**Table 6:** Comparisons between pairs of clusters for men for the variables included in the SOM analysis.

Men		Cluster					
		1					
**Cluster**	**2**	<Age					
		<Grip/BMI	**Cluster**				
		>Fat%	**2**				
	**3**	<Grip/BMI					
		<LAM%	<LAM%	**Cluster**			
		>Fat%	>Fat%	**3**			
	**4**	>Age	>Age	>Age			
		<Gait/height	<Gait/height	<Gait/height			
		<Grip/BMI					
		<Balance	<Balance	<Balance			
		<LAM%		>LAM%	**Cluster**		
				<Fat%	**4**		
	**5**		>Age		<Age		
		<Gait/height			>Gait/height		
		<Grip/BMI	<Grip/BMI	<Grip/BMI	<Grip/BMI		
		<Balance	<Balance	<Balance			
		<LAM%	<LAM%		<LAM%	**Cluster**	
		>Fat%	>Fat%		>Fat%	**5**	
	**6**	>Age	>Age	>Age		>Age	
		<Grip/BMI	<Grip/BMI	<Grip/BMI	<Grip/BMI		
					>Balance	>Balance	
		<LAM%	<LAM%		<LAM%		**Cluster**
		>Fat%	>Fat%		>Fat%		**6**
	**7**		>Age	>Age			
		<Gait/height	<Gait/height	<Gait/height		<Gait/height	<Gait/height
		<Grip/BMI	<Grip/BMI	<Grip/BMI	<Grip/BMI	<Grip/BMI	
		<Balance	<Balance	<Balance			<Balance
		<LAM%	<LAM%		<LAM%		
		>Fat%					

Means comparisons were applied to gait speed/height (Gait/Height), grip strength/body mass index (Grip/BMI), one-legged stance (Balance), lean appendicular mass percentage (LAM%), and fat percentage (Fat%). Only statistically significant results are shown (*p* < 0.05). Each result should be read as follows: The VARIABLE mean for men in Cluster ROW is greater/less (>/<) than the VARIABLE mean for men in Cluster COLUMN. For example: The age mean for men in Cluster 2 is less than the age mean for men in Cluster 1.

**Table 7:** Comparisons between pairs of clusters for men for conditions not included in the SOM analysis.

Men		Cluster					
		1					
**Cluster**	**2**						
			**Cluster**				
			**2**				
	**3**						
				**Cluster**			
				**3**			
	**4**		12.6 PVD				
		5.0 HTN	6.7 HTN	4.7 HTN	**Cluster**		
		1.8 NUMCOM	3.1 NUMCOM	1.9 NUMCOM	**4**		
	**5**		10.0 PVD				
		3.5 HTN	4.7 HTN	3.2 HTN		**Cluster**	
			2.4 NUMCOM			**5**	
	**6**	5.3 PVD	30.0 PVD	7.2 PVD			
		2.0 NUMCOM	3.4 NUMCOM	2.1 NUMCOM			**Cluster**
							**6**
	**7**		12.8 HTN				
		2.2 NUMCOM	3.7 NUMCOM	2.3 NUMCOM			

Significant (*p* < 0.05) relative risk ratios when comparing pairs of clusters. Univariate multinomial logistic regression was applied to peripheral vascular disease (PVD) and hypertension (HTN). Poisson regression applied to number of comorbidities (NUMCOM). Each result for the dichotomic variables should be read as follows: Men in ROW Cluster are RRR times more/less (if RRR > 1/if RRR < 1) likely to present CONDITION, than men in COLUMN Reference Cluster. For example: Men in Cluster 4 are 5.0 times more likely to present HTN than men in Cluster 1.

## Data Availability

Databases are anonymized and available as Supplementary Material named database.csv.

## Supplementary Materials

The following supporting information can be downloaded at: https://www.mdpi.com/article/10.3390/ijerph191912412/s1, CSV File: database.xlsx–Database used for the analysis.

## Appendix A

Neural networks that use an unsupervised training algorithm are useful for knowledge discovery in databases. In our case, the SOM Neural network was instrumental in discovering and characterizing the multidimensional profiles of an adult population.

The SOM neural network comprises two layers of neurons: an input layer with as many neurons as the number of variables used to characterize the profiles to be analyzed and an output layer, which is a hexagonal grid of neurons in a 2D space. According to the mathematical model we use to represent profiles, the data set belongs to a 6D space, each space dimension corresponding to one of the variables that characterize the older adults’ profiles. Every neuron is associated with a profile vector belonging to the input data space.

During the training phase, all the profile data (which are vectors in multidimensional space) are iteratively presented to the input layer of the neural net, triggering an adaptive process by which the output layer neurons “compete” to determine a winning neuron (hexagon) to which the profile data will be assigned. In each iteration, the winning neuron will be the one whose associated profile is most similar to the presented data profile. The similarity is calculated using the Euclidian distance among points in 6D space (square root of the sum of the squared differences between the six considered variables). This metric is adequate in our case because there are no constrictions imposed on the data set. During training with each iteration, the weight vectors associated with the neurons are adjusted to more closely resemble data. Once the training process ends, all the input data are distributed in the output layer, in such a way that neurons close in this neural plane grid will receive data with similar profiles.

The hexagonal grid of neurons that constitutes the output layer is colored to create visual scenarios: knowledge maps. In this paper, we use two visual scenarios called Clusters maps and Heat maps, which are useful for results interpretation.

LabSOM, the software tool we use, trains the neural net and incorporates Vesanto’s methodology [68] to create a Clusters map, employing an agglomerative hierarchical clustering algorithm executed over the output layer’s weights as. LabSOM assigns the same color to the neuron’s hexagons belonging to the same cluster.

Each cluster in the clusters map represents an individual profile of the set of older adults. To characterize each of these physical profiles, we display a set of six heat maps that allows us to visually interpret the meaning of each location in the cluster map. These heat maps are colored according to a chromatic scale, assigning the color red to those map’s zones where the worst variable values appear, green to the best, and yellow to intermediate values [68].

## Appendix B

Differences between means and distributions of the variables of the neural network analysis.

- One-way Welch ANOVA and Games–Howell post hoc test for the variables that were normal but variances are not homogenous.
- One-way ANOVA and Tukey post hoc test for the variables that were normal with homogeneous variances.
- Kruskal–Wallis and Dunn post hoc test for non-normal variables.

**Table A1:** Differences between means and distributions of the variables of the neural network analysis for women.

Women		Age **	Gait/height *	Grip/BMI *	Balance **	LAM% *	Fat% **
Cluster vs. Cluster		*p*-Value	*p*-Value	*p*-Value	*p*-Value	*p*-Value	*p*-Value
**1**	**2**	0.480	0.772	0.001	1.000	<0.001	<0.001
	**3**	0.532	0.023	<0.001	1.000	<0.001	<0.001
	**4**	0.749	0.040	<0.001	<0.001	<0.001	<0.001
	**5**	<0.001	0.765	<0.001	1.000	0.004	0.086
	**6**	<0.001	<0.001	0.001	<0.001	1.000	1.000
	**7**	<0.001	<0.001	<0.001	<0.001	<0.001	<0.001
**2**	**3**	1.000	0.003	<0.001	1.000	<0.001	<0.001
	**4**	1.000	0.064	0.001	<0.001	<0.001	<0.001
	**5**	<0.001	1.000	<0.001	0.003	0.005	0.340
	**6**	<0.001	<0.001	0.261	<0.001	<0.001	0.002
	**7**	<0.001	<0.001	<0.001	<0.001	<0.001	<0.001
**3**	**4**	1.000	1.000	0.041	<0.001	1.000	1.000
	**5**	<0.001	0.003	0.991	0.019	<0.001	<0.001
	**6**	<0.001	0.006	0.486	<0.001	<0.001	<0.001
	**7**	<0.001	<0.001	<0.001	<0.001	0.002	0.012
**4**	**5**	<0.001	0.065	0.009	<0.001	<0.001	<0.001
	**6**	<0.001	0.027	1.000	1.000	<0.001	<0.001
	**7**	<0.001	<0.001	<0.001	1.000	0.041	0.729
**5**	**6**	0.976	<0.001	0.301	<0.001	0.012	0.167
	**7**	0.010	<0.001	0.008	<0.001	<0.001	<0.001
**6**	**7**	1.000	0.979	0.011	1.000	<0.001	<0.001

* Welch ANOVA, Games–Howell post hoc test. ** Kruskal–Wallis, Dunn post hoc test.

**Table A2:** Differences between means and distributions of the variables of the neural network analysis for men.

Men		Age *	Gait/height *	Grip/BMI *	Balance ***	LAM% ***	Fat% **
Cluster vs. Cluster		*p*-Value	*p*-Value	*p*-Value	*p*-Value	*p*-Value	*p*-Value
**1**	**2**	0.030	0.489	<0.001	1.000	1.000	<0.001
	**3**	0.739	0.732	<0.001	1.000	<0.001	<0.001
	**4**	<0.001	<0.001	<0.001	<0.001	0.042	0.052
	**5**	0.850	0.008	<0.001	<0.001	<0.001	<0.001
	**6**	<0.001	0.156	<0.001	1.000	<0.001	<0.001
	**7**	0.232	<0.001	<0.001	<0.001	<0.001	0.021
**2**	**3**	0.542	0.999	0.976	1.000	<0.001	<0.001
	**4**	<0.001	<0.001	0.999	<0.001	1.000	0.950
	**5**	<0.001	0.753	0.007	<0.001	<0.001	<0.001
	**6**	<0.001	0.951	<0.001	1.000	<0.001	0.011
	**7**	0.001	0.001	<0.001	0.008	<0.001	0.076
**3**	**4**	<0.001	<0.001	1.000	<0.001	0.001	<0.001
	**5**	0.076	0.328	0.049	<0.001	1.000	0.974
	**6**	<0.001	0.775	<0.001	1.000	1.000	1.000
	**7**	0.029	<0.001	<0.001	<0.001	0.8391	0.426
**4**	**5**	<0.001	0.003	0.012	1.000	0.001	<0.001
	**6**	1.000	0.057	<0.001	<0.001	0.002	0.003
	**7**	0.942	0.808	<0.001	1.000	0.001	0.052
**5**	**6**	0.009	1.000	0.118	<0.001	1.000	0.987
	**7**	0.674	0.012	0.001	1.000	0.783	0.339
**6**	**7**	0.961	0.030	0.480	0.012	1.000	0.504

* One-way ANOVA, Tukey post hoc test. ** Welch ANOVA, Games–Howell post hoc test. *** Kruskal–Wallis, Dunn post hoc test.

## Appendix C

Multinomial logistic regression model (univariate analysis) to determine the relationship between the cluster classification and the conditions that were not included in the neural network analysis (presence of comorbidities, cognitive impairment, dependence, depression, fear of falling, and years of education completed) and Poisson Regression to assess whether the cluster classification influences the number of comorbidities obtained on each cluster.

**Table A3:** Multinomial logistic regression model (univariate analysis) to determine the relationship between the cluster classification and peripheral vascular disease and hypertension for women.

Women		Peripheral Vascular Disease (PVD) Yes			Hypertension (HTN) Yes		
Cluster vs. Reference Cluster		*p*-Value	RRR	95% CI	*p*-Value	RRR	95% CI
**2**	**1**	0.726	1.192	(0.446–3.186)	0.267	2.113	(0.564–7.921)
**3**		0.775	1.156	(0.428–3.126)	0.056	3.606	(0.967–13.441)
**4**		0.947	0.960	(0.294–3.135)	0.045	4.452	(1.035–19.162)
**5**		0.507	0.711	(0.259–1.950)	0.002	8.095	(2.165–30.273)
**6**		0.900	1.086	(0.297–3.976)	0.084	3.967	(0.832–18.912)
**7**		0.090	2.281	(0.880–5.914)	<0.001	10.225	(2.845–36.743)
**3**	**2**	0.924	0.970	(0.514–1.830)	0.124	1.707	(0.863–3.373)
**4**		0.638	0.805	(0.327–1.985)	0.116	2.107	(0.832–5.336)
**5**		0.123	0.596	(0.309–1.150)	<0.001	3.831	(1.926–7.621)
**6**		0.862	0.911	(0.320–2.596)	0.254	1.877	(0.636–5.544)
**7**		0.025	1.913	(1.086–3.371)	<0.001	4.839	(2.635–8.887)
**4**	**3**	0.691	0.831	(0.333–2.074)	0.654	1.235	(0.492–3.101)
**5**		0.158	0.615	(0.313–1.208)	0.019	2.245	(1.141–4.416)
**6**		0.908	0.940	(0.326–2.708)	0.862	1.100	(0.375–3.226)
**7**		0.023	1.973	(1.097–3.550)	0.001	2.835	(1.564–5.142)
**5**	**4**	0.526	0.740	(0.292–1.877)	0.206	1.818	(0.720–4.588)
**6**		0.845	1.131	(0.328–3.898)	0.856	0.891	(0.256–3.102)
**7**		0.051	2.376	(0.997–5.664)	0.060	2.296	(0.964–5.470)
**6**	**5**	0.438	1.529	(0.523–4.468)	0.195	0.490	(0.166–1.443)
**7**		<0.001	3.211	(1.742–5.919)	0.447	1.263	(0.691–2.307)
**7**	**6**	0.154	2.100	(0.758–5.817)	0.072	2.578	(0.919–7.227)

RRR = Relative Risk Ratio.

**Table A4:** Multinomial logistic regression model (univariate analysis) to determine the relationship between the cluster classification and cognitive impairment and fear of falling for women.

Women		Cognitive Impairment (CI) Yes			Fear of Falling (FF) Yes		
Cluster vs. Reference Cluster		*p**-*Value	RRR	95% CI	*p**-*Value	RRR	95% CI
**2**	**1**	0.502	2.082	(0.245–17.684)	0.068	2.786	(0.925–8.385)
**3**		0.428	2.375	(0.279–20.206)	0.002	6.000	(1.949–18.472)
**4**		0.691	1.652	(0.139–19.654)	0.012	5.333	(1.453–19.579)
**5**		0.715	1.508	(0.166–13.711)	0.003	5.500	(1.781–16.987)
**6**		0.465	2.533	(0.209–30.680)	0.019	5.500	(1.331–22.734)
**7**		0.095	5.758	(0.740–44.812)	<0.001	13.696	(4.522–41.481)
**3**	**2**	0.803	1.141	(0.405–3.214)	0.022	2.154	(1.118–4.150)
**4**		0.779	0.793	(0.157–4.005)	0.169	1.915	(0.759–4.831)
**5**		0.588	0.724	(0.225–2.327)	0.044	1.974	(1.019–3.825)
**6**		0.815	1.217	(0.235–6.310)	0.220	1.974	(0.666–5.849)
**7**		0.017	2.765	(1.198–6.382)	<0.001	4.916	(2.625–9.207)
**4**	**3**	0.661	0.696	(0.138–3.519)	0.808	0.889	(0.343–2.304)
**5**		0.447	0.635	(0.197–2.046)	0.807	0.917	(0.456–1.843)
**6**		0.939	1.067	(0.205–5.545)	0.878	0.917	(0.302–2.778)
**7**		0.039	2.424	(1.046–5.620)	0.015	2.283	(1.173–4.443)
**5**	**4**	0.971	0.913	(0.165–5.036)	0.950	1.031	(0.396–2.683)
**6**		0.685	1.533	(0.194–12.092)	0.963	1.031	(0.285–3.735)
**7**		0.103	3.485	(0.779–15.642)	0.048	2.568	(1.010–6.528)
**6**	**5**	0.558	1.680	(0.297–9.512)	1.000	1.000	(0.329–3.041)
**7**		0.009	3.818	(1.407–10.359)	0.008	2.490	(1.272–4.874)
**7**	**6**	0.293	2.273	(0.492–10.505)	0.102	2.490	(0.835–7.423)

RRR = Relative Risk Ratio.

**Table A5:** Multinomial logistic regression model (univariate analysis) to determine the relationship between the cluster classification and activities of daily living dependence and years of education for women.

Women		Activities of Daily Living Dependence (ADLD) Yes			Years of Education (EDUC)		
Cluster vs. Reference Cluster		*p**-*Value	RRR	95% CI	*p**-*Value	RRR	95% CI
**2**	**1**	0.791	0.731	(0.072–7.422)	0.880	0.993	(0.904–1.091)
**3**		0.923	1.118	(0.118–10.599)	0.805	1.012	(0.920–1.113)
**4**		0.268	3.619	(0.371–35.293)	0.488	0.961	(0.859–1.075)
**5**		0.715	1.508	(0.166–13.711)	0.869	1.008	(0.916–1.110)
**6**		0.465	2.533	(0.209–30.680)	0.342	0.942	(0.834–1.065)
**7**		0.101	5.566	(0.714–43.364)	0.077	0.922	(0.842–1.009)
**3**	**2**	0.587	1.529	(0.331–7.076)	0.539	1.019	(0.959–1.084)
**4**		0.046	4.952	(1.028–23.866)	0.453	0.968	(0.890–1.054)
**5**		0.334	2.063	(0.475–8.969)	0.629	1.015	(0.954–1.080)
**6**		0.193	3.467	(0.533–22.551)	0.297	0.949	(0.861–1.047)
**7**		0.001	7.616	(2.237–25.931)	0.006	0.928	(0.880–0.979)
**4**	**3**	0.117	3.238	(0.745–14.080)	0.242	0.950	(0.871–1.035)
**5**		0.666	1.349	(0.347–5.250)	0.905	0.996	(0.934–1.062)
**6**		0.369	2.267	(0.380–13.537)	0.160	0.931	(0.843–1.029)
**7**		0.004	4.980	(1.674–14.811)	<0.001	0.911	(0.861–0.963)
**5**	**4**	0.222	0.417	(0.102–1.697)	0.282	1.049	(0.962–1.144)
**6**		0.701	0.700	(0.113–4.329)	0.737	0.981	(0.874–1.100)
**7**		0.462	1.538	(0.489–4.840)	0.304	0.959	(0.885–1.039)
**6**	**5**	0.558	1.680	(0.297–9.512)	0.186	0.935	(0.846–1.033)
**7**		0.011	3.691	(1.357–10.036)	0.002	0.914	(0.863–0.968)
**7**	**6**	0.314	2.197	(0.475–10.170)	0.640	0.978	(0.890–1.074)

RRR = Relative Risk Ratio.

**Table A6:** Poisson Regression to determine the relationship between the cluster classification and the number of comorbidities for women.

Women		Number of Comorbidities (NUMCOM)		
Cluster vs. Reference Cluster		*p**-*Value	RRR	95% CI
**2**	**1**	0.898	1.025	(0.701–1.499)
**3**		0.510	1.136	(0.777–1.663)
**4**		0.191	1.333	(0.866–2.053)
**5**		0.253	1.248	(0.854–1.823)
**6**		0.438	1.212	(0.745–1.971)
**7**		0.004	1.686	(1.180–2.408)
**3**	**2**	0.395	1.109	(0.874–1.406)
**4**		0.100	1.301	(0.951–1.779)
**5**		0.102	1.217	(0.962–1.541)
**6**		0.393	1.182	(0.805–1.737)
**7**		<0.001	1.644	(1.350–2.003)
**4**	**3**	0.318	1.173	(0.858–1.605)
**5**		0.438	1.098	(0.867–1.391)
**6**		0.743	1.067	(0.726–1.568)
**7**		<0.001	1.483	(1.217–1.808)
**5**	**4**	0.677	0.936	(0.685–1.278)
**6**		0.668	0.909	(0.588–1.406)
**7**		0.106	1.264	(0.952–1.679)
**6**	**5**	0.882	0.971	(0.662–1.426)
**7**		0.003	1.351	(1.111–1.643)
**7**	**6**	0.074	1.391	(0.969–1.997)

RRR = Relative Risk Ratio.

**Table A7:** Multinomial logistic regression model (univariate analysis) to determine the relationship between the cluster classification and peripheral vascular disease and hypertension for men.

Men		Peripheral Vascular Disease (PVD) Yes			Hypertension (HTN) Yes		
Cluster vs. Reference Cluster		*p**-*Value	RRR	95% CI	*p**-*Value	RRR	95% CI
**2**	**1**	0.121	0.175	(0.019–1.585)	0.653	0.742	(0.202–2.724)
**3**		0.640	0.729	(0.194–2.739)	0.909	1.069	(0.342–3.344)
**4**		0.180	2.211	(0.693–7.051)	0.005	4.987	(1.631–15.252)
**5**		0.365	1.750	(0.522–5.867)	0.031	3.455	(1.119–10.669)
**6**		0.037	5.250	(1.107–24.905)	0.255	2.375	(0.535–10.534)
**7**		0.912	0.875	(0.082–9.376)	0.060	9.500	(0.913–98.803)
**3**	**2**	0.209	4.167	(0.449–38.654)	0.575	1.440	(0.402–5.157)
**4**		0.020	12.632	(1.494–106.766)	0.003	6.720	(1.915–23.577)
**5**		0.037	10.000	(1.151–86.876)	0.017	4.655	(1.315–16.475)
**6**		0.005	30.000	(2.794–322.090)	0.153	3.200	(0.649–15.775)
**7**		0.289	5.000	(0.256–97.697)	0.038	12.800	(1.149–142.577)
**4**	**3**	0.071	3.032	(0.909–10.110)	0.006	4.667	(1.571–13.866)
**5**		0.171	2.400	(0.686–8.397)	0.036	3.232	(1.077–9.703)
**6**		0.015	7.200	(1.468–35.317)	0.286	2.222	(0.512–9.647)
**7**		0.881	1.200	(0.110–13.146)	0.066	8.889	(0.866–91.199)
**5**	**4**	0.671	0.792	(0.269–2.327)	0.503	0.693	(0.236–2.030)
**6**		0.245	2.375	(0.553–10.196)	0.316	0.476	(0.112–2.031)
**7**		0.431	0.396	(0.039–3.977)	0.586	1.905	(0.188–19.326)
**6**	**5**	0.150	3.000	(0.671–13.404)	0.614	0.688	(0.160–2.955)
**7**		0.560	0.500	(0.049–5.154)	0.393	2.750	(0.270–28.036)
**7**	**6**	0.165	0.167	(0.013–2.093)	0.280	4.000	(0.323–49.596)

RRR = Relative Risk Ratio.

**Table A8:** Poisson Regression to determine the relationship between the cluster classification and the number of comorbidities for men.

Men		Number of Comorbidities (NUMCOM)		
Cluster vs. Reference Cluster		*p*-Value	RRR	95% CI
**2**	**1**	0.062	0.586	(0.335–1.028)
**3**		0.798	0.944	(0.605–1.471)
**4**		0.002	1.832	(1.248–2.689)
**5**		0.116	1.394	(0.922–2.107)
**6**		0.005	1.984	(1.224–3.217)
**7**		0.009	2.189	(1.221–3.927)
**3**	**2**	0.094	1.609	(0.923–2.807)
**4**		< 0.001	3.124	(1.876–5.200)
**5**		0.001	2.377	(1.396–4.047)
**6**		<0.001	3.383	(1.879–6.092)
**7**		< 0.001	3.733	(1.904–7.321)
**4**	**3**	0.001	1.941	(1.331–2.831)
**5**		0.061	1.477	(0.983–2.219)
**6**		0.002	2.102	(1.304–3.391)
**7**		0.004	2.320	(1.299–4.143)
**5**	**4**	0.116	0.761	(0.541–1.070)
**6**		0.711	1.083	(0.710–1.653)
**7**		0.514	1.195	(0.700–2.041)
**6**	**5**	0.124	1.424	(0.908–2.232)
**7**		0.112	1.571	(0.900–2.741)
**7**	**6**	0.752	1.103	(0.599–2.032)

RRR = Relative Risk Ratio.
